# Bayesian Convolutional Neural Networks in Medical Imaging Classification: A Promising Solution for Deep Learning Limits in Data Scarcity Scenarios

**DOI:** 10.1007/s10278-023-00897-8

**Published:** 2023-10-03

**Authors:** Filippo Bargagna, Lisa Anita De Santi, Nicola Martini, Dario Genovesi, Brunella Favilli, Giuseppe Vergaro, Michele Emdin, Assuero Giorgetti, Vincenzo Positano, Maria Filomena Santarelli

**Affiliations:** 1 University of Pisa, Pisa, Italy; 2 Fondazione G. Monasterio CNR - Regione Toscana, Pisa, Italy; 3 Scuola Universitaria Superiore ‘S. Anna’, Pisa, Italy; 4 CNR Institute of Clinical Physiology, Pisa, Italy

**Keywords:** Bayesian convolutional neural networks, Cardiac amyloidosis, Data scarcity, Probabilistic programming, Uncertainty, Deep learning

## Abstract

Deep neural networks (DNNs) have already impacted the field of medicine in data analysis, classification, and image processing. Unfortunately, their performance is drastically reduced when datasets are scarce in nature (e.g., rare diseases or early-research data). In such scenarios, DNNs display poor capacity for generalization and often lead to highly biased estimates and silent failures. Moreover, deterministic systems cannot provide epistemic uncertainty, a key component to asserting the model’s reliability. In this work, we developed a probabilistic system for classification as a framework for addressing the aforementioned criticalities. Specifically, we implemented a Bayesian convolutional neural network (BCNN) for the classification of cardiac amyloidosis (CA) subtypes. We prepared four different CNNs: base-deterministic, dropout-deterministic, dropout-Bayesian, and Bayesian. We then trained them on a dataset of 1107 PET images from 47 CA and control patients (data scarcity scenario). The Bayesian model achieved performances (78.28 (1.99) % test accuracy) comparable to the base-deterministic, dropout-deterministic, and dropout-Bayesian ones, while showing strongly increased “Out of Distribution” input detection (validation-test accuracy mismatch reduction). Additionally, both the dropout-Bayesian and the Bayesian models enriched the classification through confidence estimates, while reducing the criticalities of the dropout-deterministic and base-deterministic approaches. This in turn increased the model’s reliability, also providing much needed insights into the network’s estimates. The obtained results suggest that a Bayesian CNN can be a promising solution for addressing the challenges posed by data scarcity in medical imaging classification tasks.

## Introduction

Artificial intelligence (AI), which is already having an impact in the field of medicine, will play an even larger role during the next few years [1]. Modern deep neural networks (DNNs) have produced remarkable achievements in data analysis, classification, and image processing. DNNs have drawn more and more the attention of experts as their involvement using medical data can improve the precision of medical applications. If large datasets are available, neural networks can interpret very complex phenomena more effectively than traditional statistical methods. Sadly, their performance is directly correlated with the size of the input [1]. This is a non-trivial criticality where datasets are scarce in nature (i.e., rare diseases or unusual/early-research data), data aggregation is not possible, and/or augmentation capabilities are limited. Deep learning models are also vulnerable to overfitting, especially when constrained by small datasets. This in turn negatively impacts their capacity for generalization [2]. This is an important challenge for situations where dramatic outcomes can result from silent failures (i.e., the network confidently failing to classify data), such as in medical diagnosis [3]. Additionally, no epistemic uncertainty, particularly significant when training data are lacking, is provided in either classification or regression use cases. Many solutions, such as dropout (during training) [4], data augmentation [5], and *k*-fold cross validation [6], have been proposed in literature to counteract overfitting and correctly assess the performance. Despite these efforts, problems regarding interpretability of the output and the related uncertainty still exist. To mitigate these issues, the Bayesian paradigm can be viewed as a systematic framework for analyzing and training uncertainty-aware neural networks, with good learning capabilities from small datasets and resistance to overfitting [7]. Particularly, Bayesian neural networks (BNNs) are a viable framework for using deep learning in contexts where there is a need to produce information capable of alerting the user if a system should fail to generalize [8]. Many studies have investigated the use of the Bayesian paradigm in medicine for classification tasks. Some applications concern the classification of histopathological images [9], oral cancer images [10], and resting state functional magnetic resonance imaging (rs-fMRI) images for Alzheimer’s disease [11]. More applications of the Bayesian paradigm are available in the thorough review work by Abdullah et al. [12].

### Bayesian Neural Networks

**Fig. 1:**
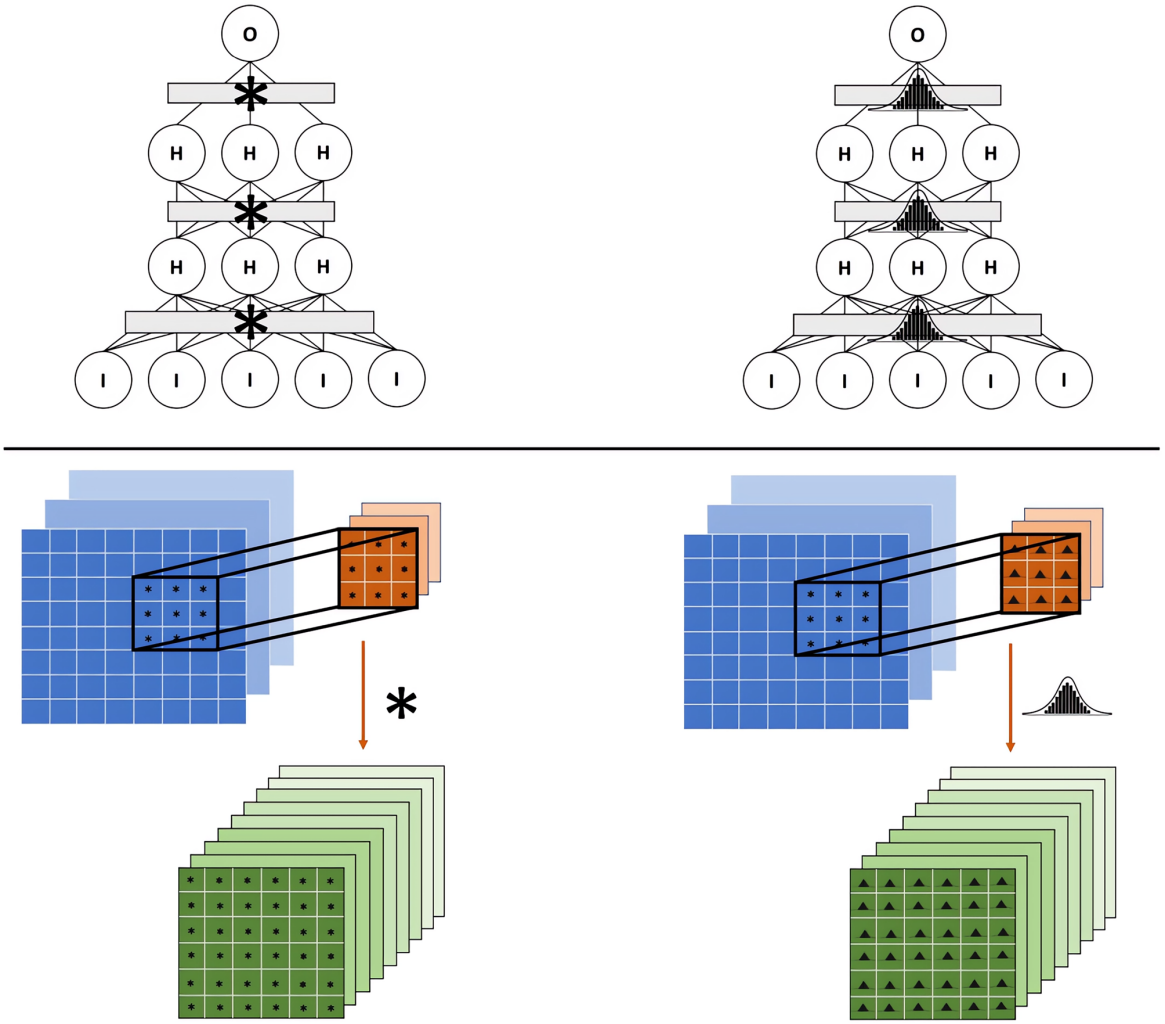
Deterministic (left) vs bayesian (right) for fully connected layers (up) and convolutional layers (down)

The concept behind BNNs comes from the application of the Bayesian paradigm to artificial neural networks (ANNs) in order to render them probabilistic systems. The Bayesian approach to probability (in contrast to the frequentist approach) spans from the meaning behind Bayes’s rule shown in the Eq. 1:

1 $$\begin{aligned} P(H|D) = \frac{P(D|H)P(H)}{P(D)} \,, P(D) = \int P(D|\theta ) P(\theta ) \,d\theta \end{aligned}$$

where *P*(*H*|*D*) is called the *posterior*, *P*(*D*|*H*) the *likelihhod*, *P*(*H*) the *prior*, and *P*(*D*) the *evidence*. *P*(*D*) is obtained by integrating over all the possible parameter in order to normalize the *posterior*. This step is intractable for practical models and is tackled through various approaches (see also *predictive* *posterior* later). *H* and *D* respectively represent the hypothesis and the available data. Applying the Bayes’ formula to train a predictor can be thought of as learning from data *D* [8]. One possible description for a BNN is that of a stochastic neural network trained using Bayesian inference [8]. The design and implementation of a BNN is compound of two steps: the definition of the network architecture and the selection of a stochastic model (in terms of prior distribution on the network’s parameters and/or prior confidence in the predictive capabilities) [8]. The stochastic part in model parametrization can be viewed as the formation of the hypothesis *H* [8]. Looking at the Eq. 1 also gives a more complete picture of the probabilistic point of view for the training process. Initially, the *prior* is defined during the network’s construction process. We then proceed at the computation of the *likelihood* (how good the model fits the data) through some form of probabilistic alternative to forward and back-propagation. Lastly, we normalize the result for the *evidence* (all the possible models fitting the data) in order to update our prior belief with new found information and construct the new *posterior*. This process is repeated throughout various epochs, as for classic neural networks, until performance criteria are met. Epistemic uncertainty is included in the *posterior* [8] during training and at inference. More precisely, once the model is trained, at inference time, an approximate form of the *predictive* *posterior*, of which the analytical form is shown in Eq. 2, is used.

2 $$\begin{aligned} P(\hat{y}|\hat{x},D) = \int P(\hat{y}|\hat{x},\theta )P(\theta |D) \,d\theta \end{aligned}$$

where $P(\hat{y}|\hat{x},D)$ represents new data probability given the known data, $P(\hat{y}|\hat{x},\theta )$ represents the probability with respect to model parameters, and it considers the effect the known data have on the parameters ($P(\theta |D)$). This means that, with the same stochastic model and equal inputs, different outputs can be given, cumulatively providing an epistemic uncertainty profile. True Bayesian inference for large neural networks is intractable (integrals on milions of parameters for evidence and *predictive* *posterior*), so alternative methods, such as variational inference [13], Markov Chain Monte Carlo [14], and dropout Bayesian approximation [15], are used in order to render these models computationally feasible. Giving more insight in the world of BNNs is not in the scope of this article, but good resources are available in the literature such as Jospin et al. [8] and Mullachery et al. [16]. In this work, we propose a Bayesian convolutional neural network (BCNN), a convolutional neural network (CNN) in structure with normal distributions imposed on parameters as *priors* for stochastic model parametrization (Fig. 1). The CNN underlying architecture follows the traditional structure, with convolutional layers to extract the input’s features and subsequent fully connected layers to proceed with the classification. From this, the BCNN is obtained simply by using Bayesian layers instead of traditional ones, as better explained in the “Materials and Methods” section. The BCNN will be trained with variational inference [13] through back-propagation using Bayes-by-backprop [17] with the local reparametrization trick [18]. Another type of approximate BCNN will be proposed through dropout-Bayesian approximation [15], as a more computationally light method.

### The Diagnosis of Cardiac Amyloidosis

Amyloidosis are a class of disorders caused by the extracellular deposition of soluble misfolded proteins that collect and deposit as amyloid fibrils [19]. The heart, as of many other organs, is affected by this disease and frequently involved, particularly in immunoglobulin light chain amyloidosis (AL) and transthyretin-related amyloidosis (ATTR) [20]. These two subtypes of amyloidosis call for different treatments: while ATTR patients receive small RNA-silencing molecules or stabilizers, AL patients are typically treated with chemotherapy or stem cell transplantation [21, 22]. Additionally, cardiac amyloidosis (CA) can be frequently misdiagnosed, particularly in its early stages, resulting in a significant diagnosis delay, which may reduce the efficacy of the treatments [23]. The characterization of CA by PET imaging, particularly following [18F]-florbetaben injection, has recently gained momentum in the literature [24, 25]. According to Kim et al. [25], this diagnostic methodology has a sensitivity of 0.95 (0.87–0.99) and a specificity of 0.98 (0.87–1.00) when discerning AL-CA patients from non AL-CA. Typically, one or two 3D PET images are acquired between 40 min and 1 h following the injection of the tracer, and they are visually examined in relation to standardized uptake values (SUV). The examination could be greatly optimized if an appropriate diagnosis of CA could be made from images obtained quickly, i.e., just a few minutes after the radiotracer injection [23]. Unfortunately, early PET acquisitions have not yet been shown to be able to diagnose the existence of cardiac amyloidosis, and late acquisitions are still not capable of accurately discerning between ATTRs and CTRLs [26]. Ideally, we would like to be able to correctly diagnose the different subtypes of CA through images acquired early, without the need to compute derived values, so that both the acquisition and diagnostic pipeline could be optimized.

### The Clinical Study

In this work, to explore the potential in adopting the Bayesian paradigm, we present the workflow regarding the development of a BCNN for cardiac amyloidosis (CA) subtype classification from PET images acquired 15 min after [18F]-florbetaben injection. We will then compare it to a traditional deterministic CNN, a dropout-deterministic and a dropout-Bayesian one (with all of the mentioned CNN’s sharing the same architecture). The intention is to provide the application of the proposed method for rare or quasi-rare pathologies diagnosis in nuclear medical imaging, where much of the problems previously highlighted represent a typical situation due to low data availability. The objective is the construction of an uncertainty aware classification system able to produce reliable results and give insight necessary for future application in clinical practice. Moreover, such analyses are performed on early acquired PET images, not requiring any derived value computation.

## Matherials and Methods

### Subjects’ Images Acquisition and Preprocessing

Forty-seven subjects, acquired from 2016 to 2020 in the Nuclear Medicine Unit of Fondazione Toscana Gabriele Monasterio (FTGM), were used in this retrospective study (13 ATTR-CA patients, 15 AL-CA patients, and 19 control patients). Following the results given by the first 6 patients (2 AL, 2 ATTR, 2 CTRL) the sample size was estimated at 9 for each group, given a power of 0.95 and a small effect (G*Power Software, version 3.1, University of Dusseldorf Department of Psychology, Dusseldorf, Germany). The controls had a CA-like suspicion which ended up with a different diagnosis (such as hypertensive cardiac hypertrophy, primary hypertrophic cardiomyopathy, or left ventricular hypertrophy secondary to an aortic valve). According to the most recent cardiological evidence and guidelines [27, 28], the diagnosis of CA was made by combining multiple clinical investigations: clinical examination, biomarkers positivity (N terminal fraction of pro-brain natriuretic peptide, high sensitivity troponin T, immunoglobulin light-chains in serum and/or in urine), electrocardiogram, echocardiography, bone-scintigraphy, CMR and histology of amyloid deposition. None of the ATTR subjects had serum or urinary monoclonal component. Note that the final label was assigned through cardiac biopsy, so the diagnosis is to be considered certain. Both the AIFA (Agenzia Italiana del Farmaco) committee and the institutional ethics committee gave their approval to the study. The research complied with the Helsinki Declaration. An informed consent form was signed by all the participants. PET/CT images were acquired using a Discovery RX VCT 64-slice tomography (GE Healthcare, Milwaukee, WI, USA). The heart was first imaged using a low-dose computed tomography (CT). Then, for roughly 40 min, PET acquisition in list mode was carried out. The [18F]-florbetaben intravenous bolus injection signaled the beginning of the PET acquisition. A sinogram was created from the raw list-mode data that covered a time range of 5 min, beginning 15 min after the injection. Then, using the ordered-subset expectation maximization (OSEM) iterative technique, PET pictures were rebuilt to provide 3D static images. Forty-seven axial slices with a 128×128 pixel matrix made up each 3D volume. Of these, only those covering the heart were used in the investigation, which resulted in a range of 21 to 25 slices, on average, being taken into consideration for each patient. The selected images were subsequently cropped, obtaining 77×104 pixels heart-centered slices. After the pre-processing steps, 1107 images (375 AL, 312 ATTR, and 420 CTRL) were selected. These were divided in 2 groups of 38 and 9 subjects. To avoid data leakage, we used the first group (10 ATTR, 12 AL, 16 CTRL) for training and validating the network (80% training, 20% validation) and the second group (3 ATTR, 3 AL, 3 CTRL) for testing; the same datasets were used for all the developed models. The training, validation, and test set comprised of 717, 180, and 210 images respectively. 5x data augmentation was used, through image transformations composed randomly of ± 10° rotations and ± 10% horizontal and vertical translations, producing 3585 new images, for a total of 4302 images for the training set. Images were previously rescaled from 16 bit to double precision photon coincidence counting and then subsequently normalized to values between 0 and 1 (float32) in order to accelerate convergence. Labels were one-hot encoded for the three classes.

### Networks’ Architecture

**Fig. 2:**
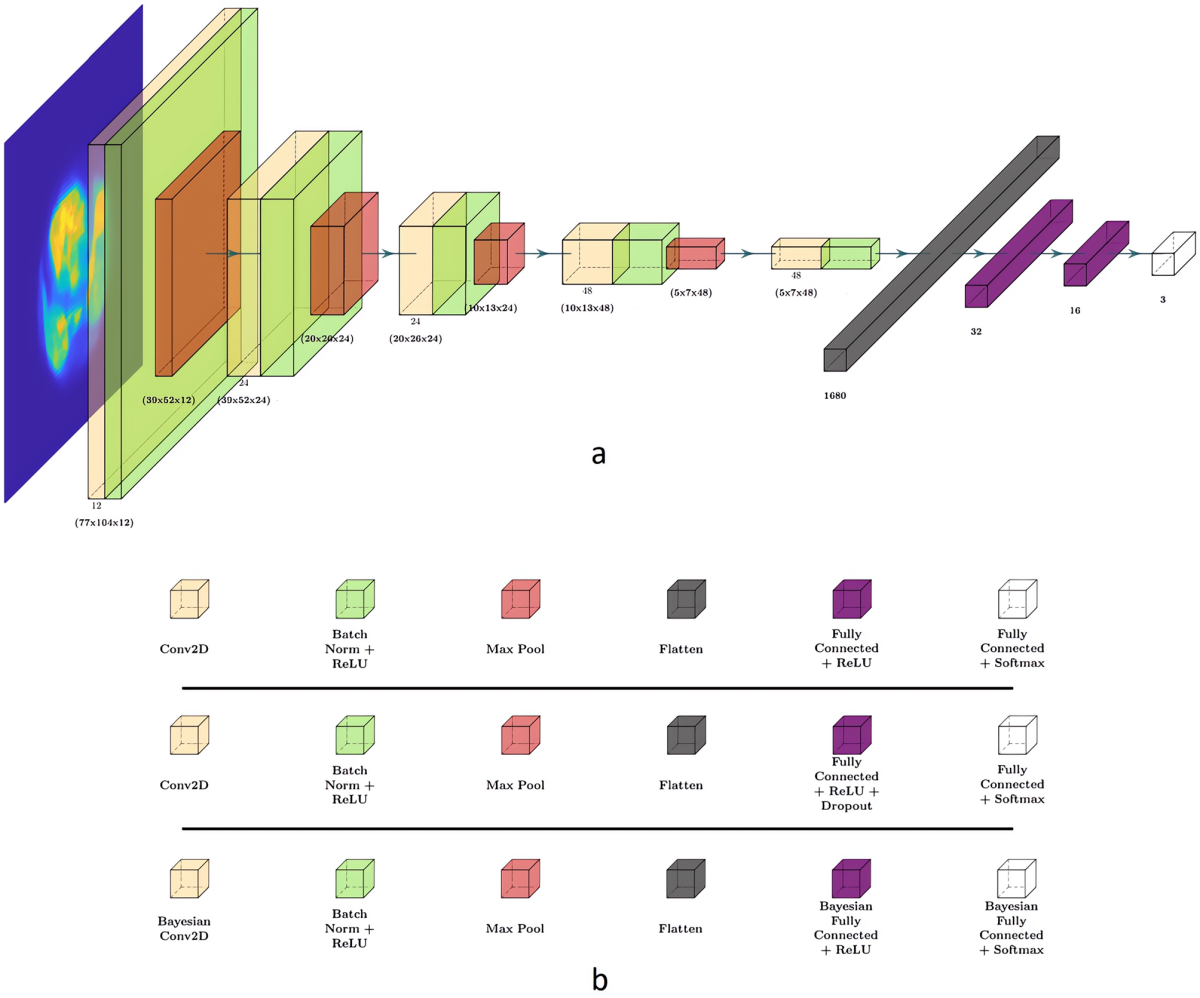
Networks’ architecture (**a**) and legends (**b**) for CNN (up), DropCNN and DropBCNN (mid) and BCNN (down)

The architecture for the CNN, dropout-deterministic CNN, (DropCNN), dropout-Bayesian CNN (DropBCNN), and BCNN is comprised of 5 convolutional modules and a final classifier made of 3 linear layers with respective ReLU activation functions (except in the last layer, where the ReLU is substituted with a Softmax to obtain probabilities from logits). Each convolutional module is made up of a convolutional layer of 12 filters (each 3×3), a batch normalization (in order to help with the network’s regularization), a ReLU, and a final max pooling layer of dimension 3×3. Padding for the convolutional layers was set to “same” to maintain image dimension; padding for the max pooling layers was set to 1 in both *x* and *y* dimensions. In the last convolutional module, the max pooling is substituted with a flattening layer in order to proceed with the classification in the final layers of the network. The difference between the four networks arise in the type of convolutional and classification layers used. While in the BCNN both the convolutional and linear layers are Bayesian and based on Gaussian mixture priors, for the DropBCNN, DropCNN and CNN, the layers are classic deterministic, and the DropBCNN and DropCNN have dropout layers after the first two dense layers. The network has 93,827 parameters for the deterministic and dropout implementations. For the BCNN, 312 parameters are point estimates, and 93,515 are drawn from distributions. Considering the approach used for the Bayesian layers described in Blundell et al. [17], the total number of parameters in the BCNN is then raised to 187,342 (parameters are doubled for all the wheights drawn form a distribution). The schematic common to all the networks is shown in Fig. 2 with the relative legends.

### Networks’ Implementation

We developed the architecture in Python, using the PyTorch framework [29] and Blitz [30] library for the Bayesian layers. Starting from the handcrafted datasets, we proceeded to split the training and validation dataset according to a principle of iterative stochastic validation. As a first step, we performed tuning of the hyperparameters on a specific split of the training and validation dataset. Then, we validated the network by repeating the training and validation steps while random splitting the dataset each time (five times). Finally, we considered the validation performance as a mean of all the results obtained with this method. We used a batch size of 128 images for each iteration (34 iterations per epoch) for a total of 125 epochs with a $1.25*10^{-5}$ learning rate, using Adam optimizer with default parameters and cross-entropy loss. For the Bayesian layers, we used a double Gaussian mixture prior with mean equal to 0, first standard deviation equal to 1, second standard deviation equal to 0.5, and mixture weight equal to 0.5 (meaning the second prior weights half the first). For both the validation and training sets’ loss and accuracy evaluation (and, consequently, for the backpropagation algorithm), we sampled 3 Monte Carlo estimates of the gradient and mediated the results. A synthesis of the used hyperparameters for the models is available in Table 1. The CNN with dropout layers was validated and tested using dropout only during training (as it is normally done, in this case is referred to as DropCNN) and as an approximate Bayesian network by keeping dropout active during evaluation and test (in this case is referred to as DropBCNN). The final test results for the DropBCNN and BCNN were obtained by sampling 100 deterministic models from the trained networks and assigning the class by majority voting of the sampled population. The training was carried out on a system with a 6-cores/12threads Intel i7 7800X CPU, 64 GB of RAM, GTX 1080Ti GPU, and Ubuntu 22.04 LTS OS. Each training epoch took $\sim$1 s for the CNN, DropCNN, and DropBCNN, while the BCNN required $\sim$ 6.5 s (reducible to 4.5 s by only taking one Monte Carlo estimate of the gradient). Classifying an image required $\sim$1.9 ms for the CNN, DropCNN and DropBCNN and $\sim$9.5 ms for the BCNN. Note that, to obtain a useful classification with the corresponding uncertainty profiles, the probabilistic networks need to classify an image for *n* different times and then vote by majority, so the time for the DropBCNN and BCNN should be considered *n* times (*n* = 100 in our case).

**Table 1:** Hyperparameters for the proposed networks

**Hyperparameter**	**Value**
Number of epochs	125
Learning rate	$1.25*10^{-5}$
Batch size	128
Optimizer	*Adam*
Loss function	*CrossEntropyLoss*
Prior (only BCNN)	Normal mixture (*mean* = 0, *SDs* = [1, 0.5], *weight* = 0.5)
Monte Carlo gradient estimates (only BCNN)	3

## Results

**Fig. 3:**
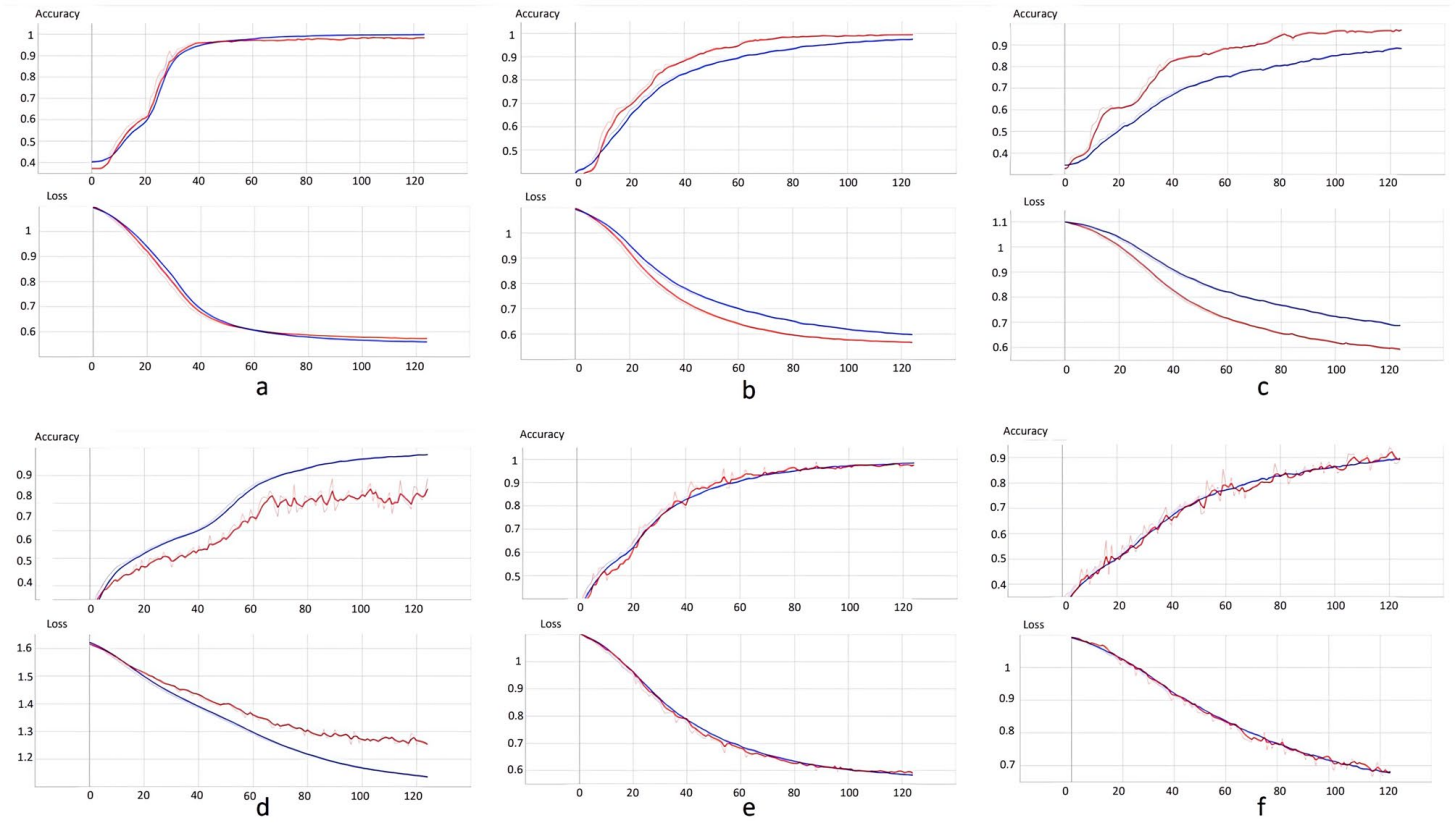
Learning curves (blue for training and red for validation) for the CNN (**a**), DropCNN with *p* = 0.25 (**b**), DropCNN with *p* = 0.5 (**c**), BCNN (**d**), DropBCNN with *p* = 0.25 (**e**), and DropBCNN with *p* = 0.5 (**f**). The shadow lines represent the true curves, more variable due to the use of mini-batch and Bayesian methods. Full lines represent the smoothed curves, superimposed for a better interpretation of the trend

**Fig. 4:**
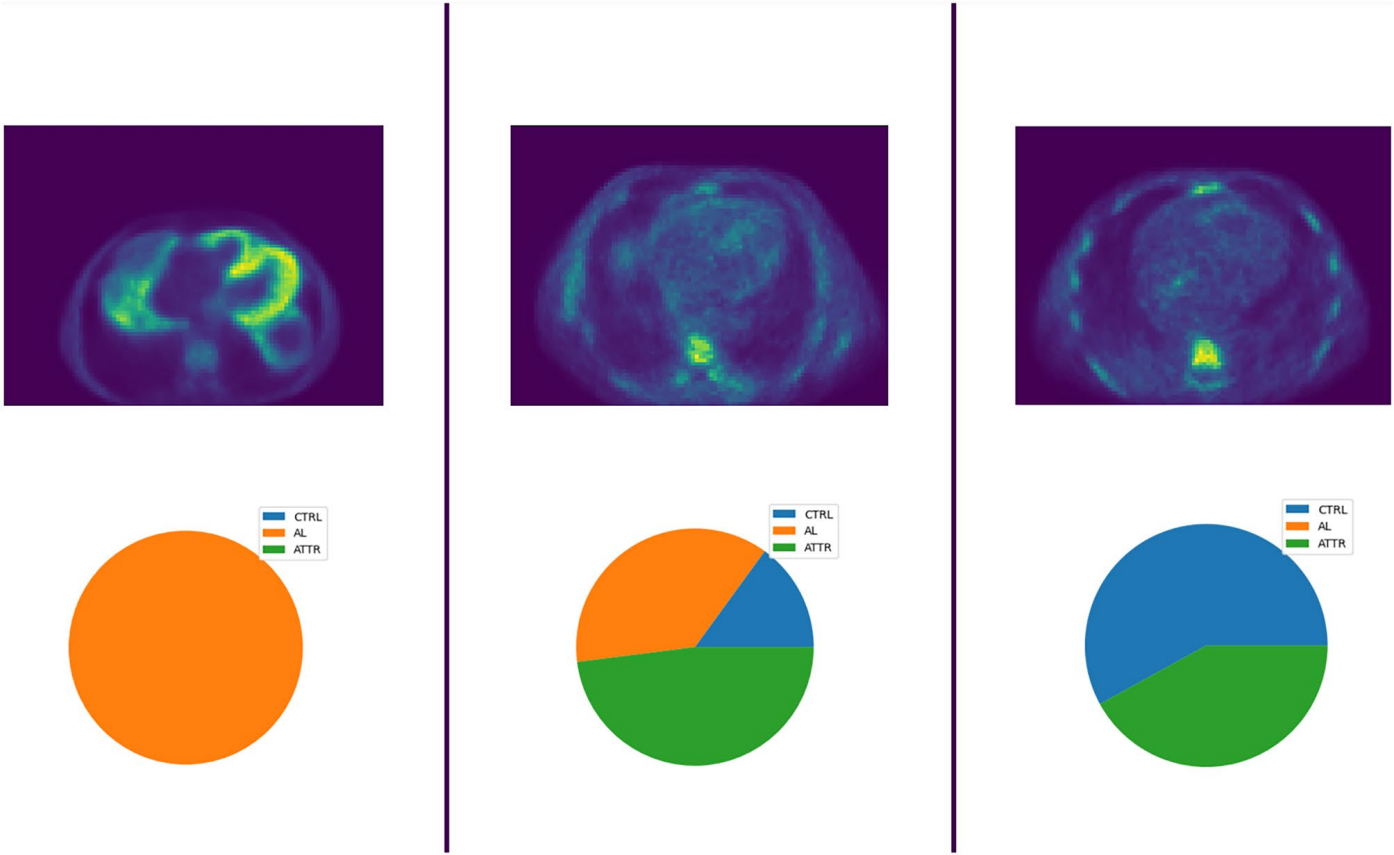
Examples of the uncertainty profiles obtained from the BCNN: AL prediction (left), ATTR prediction (center), CTRL prediction (right)

**Fig. 5:**
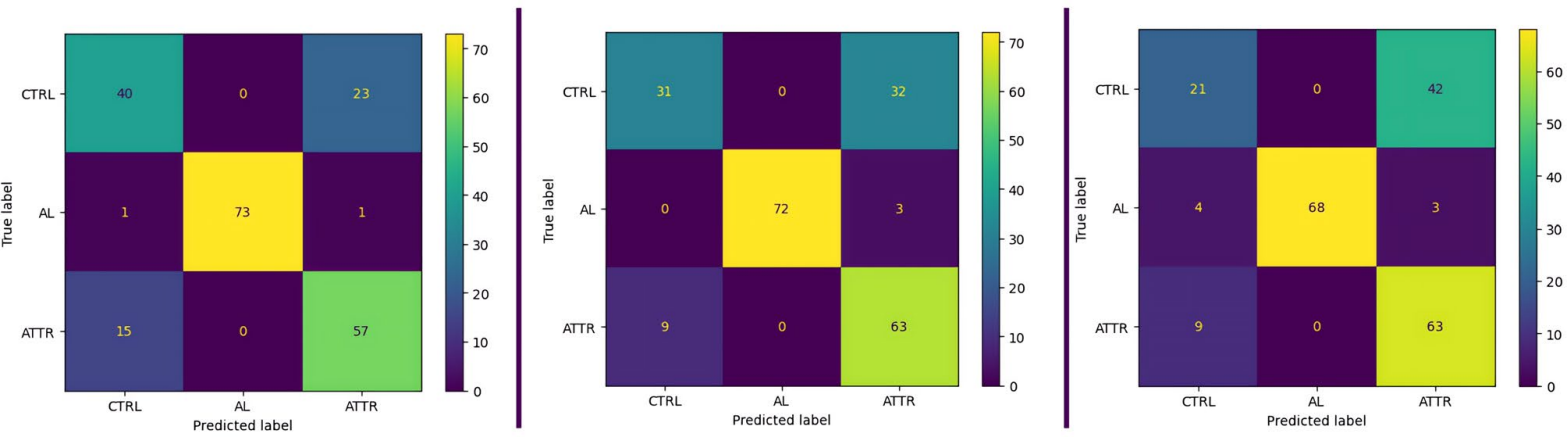
Confusion matrices: CNN (left), BCNN (center), DropBCNN with *p* = 0.5 (right)

Figure 3 shows a representative example of the learning curves for the CNN, DropCNN (dropout layers inactive at evaluation, dropout probability of 25% and 50%), DropBCNN (dropout layers active at evaluation, dropout probability of 25% and 50%), and the BCNN. Table 2 shows the result for accuracy on the four tested networks. Data are shown for accuracy on training, validation, and test set. Moreover, validation-test mismatch is provided as a measure of the capacity of the network to detect out-of-distribution (OOD) data [31]. Figure 4 shows the uncertainty profiles examples on the three different classes (those shown in the figure are relative to the BCNN). Note that these profiles are only obtainable with probabilistic instances of the network by sampling n deterministic models (*n* = 100 in this case) and considering all the resulting predictions. From this uncertainty profiles, we can gather the metrics shown in Table 6 (confidence for CTRL and ATTR was similar and is displayed as a single value). Here, the percentages refer to the number of deterministic networks (sampled from the probabilistic ones) agreeing on the inferred classification. “Correct” and “Incorrect” refer to the prediction, and “AL” and “CTRL & ATTR” refer to the corresponding true class. The CNN and DropCNN are absent from Table 6 as non probabilistic. Figure 5 shows the confusion matrices for the CNN, BCNN, and DropBCNN (with *p* = 0.5). In order to better assess the models’ performance, Tables 3, 4, and 5 report precision, recall, and F1-score respectively, in a “1 vs all” fashion.

**Table 2:** Mean (SD) accuracy values for training, validation, and test set assessed for the six networks. Last column: mean (SD) mismatch between performance on the validation and test set

**Model**	**Training Acc.**	**Validation Acc.**	**Test Acc.**	**Val-Test mismatch**
CNN	99.61 (0.24)%	98.78 (0.82)%	77.05 (2.88)%	21.73 (2.25)%
DropCNN (*p* = 0.25)	97.11 (0.60)%	98.44 (0.64)%	74.48 (1.77)%	23.93 (2.18)%
DropCNN (*p* = 0.5)	88.84 (0.96)%	97.45 (1.29)%	78.19 (3.55)%	19.26 (4.55)%
DropBCNN (*p* = 0.25)	96.97 (0.52)%	96.55 (1.29)%	75.90 (2.63)%	20.65 (2.91)%
DropBCNN (*p* = 0.5)	90.35 (1.38)%	90.11 (0.96)%	77.33 (2.14)%	13.02 (2.72)%
BCNN	96.71 (0.63)%	83.75 (2.89)%	78.28 (1.99)%	6.14 (3.83)%

**Table 3:** Mean (SD) values for precision assessed for the six networks. Metrics are evaluated in a “1 vs all” fashion

**Model**	**Precision (AL)**	**Precision (ATTR)**	**Precision (CTRL)**
CNN	99.18 (1.09)%	63.08 (2.81)%	73.68 (9.68)%
DropCNN (*p* = 0.25)	99.44 (1.13)%	59.86 (1.54)%	67.54 (5.85)%
DropCNN (*p* = 0.5)	99.48 (1.04)%	64.33 (5.57)%	74.21 (4.88)%
DropBCNN (*p* = 0.25)	99.18 (1.07)%	60.64 (2.19)%	75.50 (8.64)%
DropBCNN (*p* = 0.5)	99.72 (0.55)%	62.98 (2.85)%	72.88 (4.80)%
BCNN	97.12 (2.42)%	65.04 (5.31)%	70.98 (6.24)%

**Table 4:** Mean (SD) values for recall assessed for the six networks. Metrics are evaluated in a “1 vs all” fashion

**Model**	**Recall (AL)**	**Recall (ATTR)**	**Recall (CTRL)**
CNN	99.60 (2.72)%	85.00 (6.17)%	48.25 (6.48)%
DropCNN (*p* = 0.25)	93.87 (3.11)%	84.72 (3.40)%	39.68 (4.92)%
DropCNN (*p* = 0.5)	95.20 (4.88)%	85.00 (2.83)%	50.16 (15.82)%
DropBCNN (*p* = 0.25)	95.47 (2.32)%	88.61 (3.77)%	38.73 (2.37)%
DropBCNN (*p* = 0.5)	94.93 (2.97)%	85.27 (3.12)%	47.30 (6.61)%
BCNN	97.58 (1.77)%	77.97 (8.37)%	51.75 (14.81)%

**Table 5:** Mean (SD) values for f1-score assessed for the six networks. Metrics are evaluated in a “1 vs all” fashion

**Model**	**F1-score (AL)**	**F1-score (ATTR)**	**F1-score (CTRL)**
CNN	96.28 (1.25)%	72.28 (2.85)%	57.84 (5.57)%
DropCNN (*p* = 0.25)	96.55 (1.88)%	70.10 (1.29)%	49.79 (4.21)%
DropCNN (*p* = 0.5)	97.21 (2.40)%	73.02 (3.09)%	58.81 (11.91)%
DropBCNN (*p* = 0.25)	97.27 (1.16)%	71.99 (2.70)%	51.12 (3.84)%
DropBCNN (*p* = 0.5)	97.24 (1.56)%	72.43 (2.67)%	57.08 (5.30)%
BCNN	95.53 (2.94)%	70.11 (1.55)%	54.44 (12.32)%

**Table 6:** Mean (SD) results for uncertainty in the classification when the network is correct, incorrect, classifying ALs, and classifying CTRLs and ATTRs respectively. Mismatch between correct and incorrect predictions and between ALs and CTRLs and ATTRs predictions. The percentages refer to the confidence of the prediction

**Model**	**Correct**	**Incorrect**	**ALs**	**CTRLs & ATTRs**	**Corr. vs Incorr.**	**ALs vs CTRLs & ATTRs**
DropBCNN (p = 0.25)	95.12 (1.29)%	88.18 (0.99)%	96.34 (1.88)%	91.84 (1.88)%	6.94 (0.87)%	5.73 (1.96)%
DropBCNN (p = 0.5)	88.80 (1.93)%	81.40 (3.17)%	92.34 (2.93)%	84.19 (86.63)%	7.68 (2.61)%	8.15 (3.04)%
BCNN	89.25 (1.46)%	75.24 (5.34)%	95.97 (1.86)%	80.39 (2.77)%	14.20 (4.80)%	15.58 (3.30)%

## Discussion

The first thing to notice from the obtained results is that the use of dropout only at the training stage (DropCNN) produces a strange phenomenon resulting in higher accuracy and lower loss on the validation set compared to the training set. This can already be seen when the dropout probability is set to 25% and is exacerbated with 50% dropout probability. This should be due to the fact that, while during training only some units are active, at validation, the full feature set is used and scaled appropriately, resulting in a more robust model and sometimes higher prediction scores. For our evaluation metrics, this is non-desirable behavior, as we are taking into consideration the validation performance as an approximation of the real-world network performance on unseen patients. Treating the model as a Bayesian approximation and keeping the dropout layers active at evaluation (DropBCNN) solves this problem, effectively realigning the training and validation curves both for accuracy and loss (see Fig. 3). Moreover, although the learning curves for the BCNN seem to provide a worse picture compared to the other models, the BCNN behavior is actually the desired one in order to avoid silent failures in deep learning systems. This is visible in Table 2 where we see the strong reduction in validation-test mismatch ($\sim$7%, *p*-value < 0.05) in terms of accuracy when going to the BCNN from the DropBCNN (*p*=0.5) (Bayesian approximation) and an even stronger reduction compared to the deterministic model ($\sim$15%, *p*-value < 0.05). This is indication of the improved capability of the BCNN in learning correct features and the ability to spot OOD inputs using the same patients (of the training set) in the validation set. Not only, the BCNN is also capable of achieving comparable accuracies on the test set with respect to the deterministic CNN (see Table 2). The Bayesian models are also able to provide a measure of epistemic uncertainty as seen in Table 6 and Fig. 4. This information, not available when using deterministic networks, is invaluable to assess the reliability of the prediction, especially in medicine. Uncertainty profiles can also be used to improve the performance, give the model the capability to resist adversarial attacks [32], refuse the classification under a certain threshold to avoid failures, and guide the acquisition of more data towards where the epistemic uncertainty is the highest. Both the DropBCNN and BCNN are able to provide uncerainty metrics, but as is possible to see in Table 6, the fully Bayesian model displays a greater discrepancy both between “Correct” and “Incorrect” confidence ($\sim$7% more compared to the best DropBCNN with *p* = 0.5, *p*-value < 0.05) and between “AL” and “CTRL & ATTR” ($\sim$7% more compared to the best DropBCNN with *p* = 0.5, *p*-value < 0.05). This is in line with the confusion matrices in Fig. 5 and the metrics of precision, recall, and F1-score showing better prediction capabilities towards the AL classification vs the CTRL and ATTR discrimination for all the models (max *p*-value < 0.05). Certainly, to take into consideration is the higher computational cost of the BCNN compared to the DropBCNN and CNN. In this sense, the Bayesian approximation can be seen as a way of maintaining a measure of uncertainty while compromising between the better performance of a fully Bayesian model and the lower computational cost of a deterministic CNN.

## Study’s Limitations

The main limitation of this work lies in the specific case study (early acquired cardiac PET images from CA patients) approached with the explained methodology. In particular, in the limited dataset and in the fact that the severity of the disease was not accounted for (as a general index across the various subtypes is not available), possibly leading to biased data and dataset split. To better explore the capabilities and potentiality of the Bayesian framework in similar scenarios and to produce a severity metric based on PET acquisitions are objectives of future works. Moreover, better tuning of the models and a major exploration of possible approximations and algorithms to improve Bayesian inference performance and computational cost could also be considered future works.

## Conclusion

In the present work, four models were developed to assess, through a CA classification case study, the capability of BCNNs to overcome some of the limitations of deep learning in data scarcity scenarios. The developed BCNN showed comparable accuracy on the test dataset in comparison with the deterministic CNN; it is also able to reduce silent failures by spotting OOD inputs better than the deterministic and approximate bayesian models. Moreover, both the approximate Bayesian DropBCNN and the BCNN provided epistemic uncertainty. It is well known that epistemic uncertainty is fundamental for enriching the prediction and delivering crucial information to improve model performance, better interpret results, and possibly construct thresholds to refuse classification.

## Data Availability

Data used in this article are not available due to it being property of the health care institution.

## Code Availability

Developed code is available upon request to the corresponding author.
